# Intestinal permeability and the zonulin pathway: tight junctions, mucosal immunity, dietary modulators and clinical relevance

**DOI:** 10.3389/fnut.2026.1938370

**Published:** 2026-10-06

**Authors:** David Granizo Bermejo, Guillermo García-Pérez-de-Sevilla, Antonio Molina-López

**Affiliations:** Department of Real Madrid Graduate School, Faculty of Medicine, Health and Sports, Universidad Europea de Madrid, Campus de Villaviciosa, Madrid, Spain

**Keywords:** diet, gut barrier, intestinal permeability, microbiota, mucosal immunity, secretory IgA, tight junctions, zonulin

## Abstract

The intestinal barrier separates the luminal content, including the gut microbiota, dietary antigens and toxins, from the mucosal immune system, and its integrity is essential for immune homeostasis. Loss of barrier control, commonly termed increased intestinal permeability or leaky gut, has been implicated in a broad spectrum of gastrointestinal, metabolic, autoimmune, neuropsychiatric, hepatic and cardiovascular conditions. Paracellular transport across the epithelium is governed by apical junctional complexes, of which tight junctions are the principal regulators, while the overlying mucus layer, antimicrobial peptides, secretory immunoglobulin A and the gut-associated lymphoid tissue together constitute a multi-layered defensive interface. Among the physiological modulators of tight junctions, zonulin, identified as pre-haptoglobin-2, is the only endogenous protein currently known to reversibly regulate paracellular permeability, and its release is triggered chiefly by gliadin and by the gut microbiota. This narrative review summarizes the molecular architecture of the intestinal epithelial junctions, the immunological organization of the mucosal barrier, the zonulin signaling pathway, the principal dietary factors that compromise barrier integrity (excess glucose, fructose, fat, omega-6 fatty acids, salt, alcohol and food additives), and the clinical conditions and laboratory tests associated with intestinal permeability. A clearer understanding of the diet-microbiota-barrier-immunity axis may support nutritional strategies aimed at preserving intestinal barrier function and modulating mucosal immune activation.

## 1 Introduction

The interaction between host and environment is central to health and wellbeing, and the gastrointestinal tract is one of its most important interfaces. This is due to its very large surface area, which is continuously exposed to unknown antigens and toxic molecules; to the diversity of its microbial community; and to the complex network of mucosal immune cells that supports both innate and adaptive defense mechanisms (1, 2).

The key to intestinal immune homeostasis is the capacity of the intestinal barrier to separate luminal antigens, including part of the gut microbiota, from the mucosal immune cells. Increased epithelial permeability to food-borne allergens, pathogenic microorganisms, toxins and local inflammatory mediators, together with nutrient malabsorption, is increasingly regarded not only as a feature of acute disease but, more importantly, as a factor that raises the prevalence of many chronic inflammatory conditions, autoimmune diseases and allergies (3–5).

Barrier maintenance is achieved through both specific and non-specific mechanisms. The intestinal epithelial cells form the first physical line of host defense against potentially harmful luminal stressors, but they act in concert with a chemical barrier (mucus and antimicrobial peptides) and an immunological barrier (secretory antibodies and organized lymphoid tissue) (1, 2, 4, 6). Adjacent epithelial cells are connected by several protein structures collectively known as apical junctional complexes (tight junctions, adherens junctions and desmosomes), among which tight junctions are the most decisive for paracellular barrier function (7–11).

Despite considerable progress in describing the ultrastructure of these junctions, comparatively little is known about their physiological regulation. The discovery of zonulin, the only physiological modulator of the intercellular tight junction described to date, advanced the understanding of the mechanisms that govern intestinal permeability and led to the recognition that its up-regulation in genetically susceptible individuals may contribute to autoimmune disease (12). The aim of this narrative review is to integrate the molecular basis of the intestinal epithelial barrier, the immunological organization of the mucosa, the zonulin signaling pathway, the dietary factors that compromise barrier integrity, and the clinical conditions and diagnostic tests associated with intestinal permeability, with particular attention to the diet-microbiota-barrier-immunity axis relevant to nutritional immunology.

## 2 Architecture of the intestinal epithelial barrier

Epithelial permeability occurs through two passive routes. Transcellular permeability is the movement of ions or molecules through apical and basolateral transporters or channels across the cell membrane. Paracellular permeability is the diffusion of ions or molecules through the intercellular space, driven by the transepithelial electrochemical gradient, and is characterized by size and charge selectivity that depends largely on cell type (13). The apical junctional complex comprises three structures: tight junctions, adherens junctions and desmosomes. At the level of the tight junction the intercellular space disappears completely, whereas at the adherens junctions and desmosomes the adjacent membranes remain 15–20 nm apart (7, 9–11, 14).

The barrier function of the mucosa cannot be maintained without a well-organized network of sealing strands comprising more than 50 proteins. Tight-junction proteins fall into three groups: transmembrane proteins (occludins, claudins, junctional adhesion molecules and tricellulin), cytoplasmic scaffolding proteins (zonula occludens and cingulins) and cytoskeletal proteins (actin and myosin) (15–18). These proteins are expressed differentially along the gastrointestinal tract, a pattern that probably reflects differences in luminal exposure between intestinal segments; for example, they are expressed less in the crypts than at the villus tips, so that intercellular tightness increases from the crypt toward the apical parts of the villus (19–23). The principal transmembrane and scaffolding proteins, together with their reported clinical associations, are summarized in Table 1.

**Table 1:** Principal proteins of the intestinal tight junction and their reported clinical associations.

Protein	Type	Main function	Reported clinical association
Occludin	Transmembrane	Homophilic sealing against macromolecules; links to the cytoskeleton via ZO proteins	Reduced expression or redistribution in Crohn disease, ulcerative colitis, coeliac disease, IBS and food allergy (4, 32–38)
Claudins	Transmembrane	Principal structural component; strong homo- and heterophilic interactions; determine pore selectivity	Decreased in Crohn disease, ulcerative colitis and IBS; increased in coeliac disease (4, 24, 34–37, 45, 46)
Junctional adhesion molecules	Transmembrane	Dual role: leukocyte recruitment to inflammation sites and barrier function	Reduced expression in Crohn disease and ulcerative colitis (52, 53)
Tricellulin	Transmembrane	Seals tricellular contacts where three epithelial cells meet; recruited by angulins	Loss increases paracellular permeability *in vitro*; reduced by enteric infection (54–60)
Zonula occludens	Scaffolding	Link transmembrane junction proteins to the actin cytoskeleton; structural basis of apical complexes	Down-regulated in barrier dysfunction induced by diet and inflammation (67–69)
Cingulins	Scaffolding	Recruit scaffolding proteins to junctions and connect to actin; role still uncertain	Reduced colonic levels reported in ulcerative colitis (19, 77–84)

IBS, irritable bowel syndrome; ZO, zonula occludens.

### 2.1 Transmembrane tight-junction proteins

#### 2.1.1 Occludins

Occludins (65 kDa) were the first tight-junction proteins to be discovered, in 1993, and are central to sealing adjacent cells. Structurally they consist of an intracellular loop, two neutral extracellular loops, four transmembrane domains, a short cytoplasmic NH2-terminal domain and a long cytoplasmic COOH-terminal domain. Occludins are expressed constantly throughout the intestine and appear as distinct punctate or linear structures in the apical region of the lateral plasma membrane of villus epithelial cells. Notably, only the phosphorylated form of occludin is incorporated into the intercellular junctional complexes, whereas non-phosphorylated occludin is distributed erratically along the basolateral plasma membrane and does not contribute to barrier integrity (24, 25).

The homophilic interaction between the extracellular loops of occludins on adjacent cells appears to form a barrier against macromolecules but not against small particles. The long cytoplasmic COOH-terminal domain is required for the correct integration of occludin into the tight-junction network, and links the protein to the cytoskeleton through interaction with cytoplasmic scaffolding proteins such as zonula occludens. Reduced expression and redistribution of occludins have been observed in the intestine of patients with Crohn disease, ulcerative colitis, coeliac disease and irritable bowel syndrome (4, 26–30); intestinal biopsies from patients with a known food allergy showed lower occludin expression than those of healthy subjects upon re-exposure to the food allergen (31).

#### 2.1.2 Claudins

Claudins (27 kDa) consist of an intracellular loop, two extracellular loops, four transmembrane domains, a short cytoplasmic NH2-terminal domain and a long cytoplasmic COOH-terminal domain, and show an inconsistent expression pattern across the different segments of the gastrointestinal tract (32). Claudins appear to be the principal structural component of the tight junction (14, 33, 34): the second extracellular loop is fundamentally involved in the homophilic and heterophilic interactions between claudins, and these interactions are much stronger than those of the other tight-junction proteins (35). The long cytoplasmic COOH-terminal domain is crucial for membrane targeting of claudins to the tight-junction network. Clinically, claudin expression is decreased and redistributed in the intestine of patients with Crohn disease and ulcerative colitis, decreased and irregularly distributed in the colonic mucosa in irritable bowel syndrome (4, 27, 36–38), and conversely increased in the intestine of patients with coeliac disease (28, 38); small-intestinal biopsies from patients with food allergy exposed to allergens showed decreased claudin expression compared with healthy subjects (31).

#### 2.1.3 Junctional adhesion molecules

Junctional adhesion molecules (JAMs, 40 kDa) belong to the immunoglobulin superfamily and consist of two extracellular immunoglobulin-like domains, a transmembrane domain and a short cytoplasmic COOH-terminal domain (39). They are expressed constantly throughout the intestine and are located laterally between adjacent epithelial cells (40, 41). JAMs have a dual role: in the immune system they control the recruitment of leukocytes to sites of inflammation, while in the digestive system they contribute to barrier function (39, 42–44). This dual role makes JAMs a notable point of convergence between the structural and immunological functions of the barrier. Reduced expression of JAMs has been reported in the intestine of patients with Crohn disease and ulcerative colitis (45, 46).

#### 2.1.4 Tricellulin

Tricellulin (64 kDa) shares some structural features with occludins, consisting of an intracellular loop, two extracellular loops, four transmembrane domains, a long cytoplasmic NH2-terminal domain and a short cytoplasmic COOH-terminal domain (20, 47, 48). Whereas the transmembrane proteins described above mainly seal the intercellular space between two adjacent cells (bicellular junctions), tricellulin was the first tight-junction protein identified at the tricellular junctions where three epithelial cells meet (1, 47, 48). It is expressed differentially along the intestine (strongly in the small intestine, weakly in the large intestine) and appears as a punctate structure at the corners where three cells join (1, 49). Its tricellular localization is regulated by angulins (angulin-1, -2 and -3), transmembrane proteins that recruit tricellulin to the tricellular junctions and are themselves essential for barrier integrity (2, 50). Loss of tricellulin increases paracellular permeability to macromolecules *in vitro*, and its expression can be reduced by enteric infection (50–56).

### 2.2 Cytoplasmic scaffolding proteins

The scaffolding proteins link the transmembrane junction proteins to the actin cytoskeleton and provide the structural basis of the apical junctional complex. The zonula occludens proteins (ZO-1, ZO-2 and ZO-3) are the best characterized; they bind the cytoplasmic tails of occludins, claudins and JAMs and connect them to the actomyosin ring, so that contraction or relaxation of the cytoskeleton is transmitted to the junction (57–59). Cingulins recruit further scaffolding proteins to the junction and also connect to actin, although their precise role in barrier function remains uncertain; reduced colonic levels of cingulin have been reported in ulcerative colitis (60–67). Down-regulation of zonula occludens proteins is a common feature of barrier dysfunction induced by diet and inflammation and is one of the molecular endpoints through which the dietary factors discussed below compromise the barrier.

### 2.3 Adherens junctions and desmosomes

Adherens junctions initiate and stabilize cell–cell contacts and are formed by transmembrane proteins (cadherins and nectins), cytoplasmic scaffolding proteins (catenins and afadins) and cytoskeletal proteins (9). Desmosomes are lateral patch-like junctions that anchor intermediate filaments and join adjacent cells; they comprise transmembrane proteins (desmogleins and desmocollins), scaffolding proteins (plakophilins, plakoglobins and desmoplakins) and intermediate filaments (68–70). Although they do not seal the paracellular space as tightly as the tight junctions, adherens junctions and desmosomes provide the mechanical cohesion required for the epithelium to withstand the continuous mechanical stress of the intestinal lumen, and their disassembly precedes and helps tight-junction opening.

### 2.4 The chemical barrier: mucus and antimicrobial peptides

Above the epithelial cells, a chemical barrier limits direct contact between luminal microorganisms and the apical membrane. Goblet cells secrete mucins, principally MUC-2, that polymerize into a hydrated gel forming two layers in the colon: a dense inner layer that is normally kept almost free of bacteria, and a looser outer layer colonized by the microbiota. Paneth cells, located at the base of the small-intestinal crypts, secrete antimicrobial peptides such as defensins, lysozyme and regenerating islet-derived proteins, which shape the composition of the microbiota and protect the stem-cell niche. Several of the dietary factors discussed in this review (notably alcohol and emulsifiers) reduce MUC-2 expression and thin the mucus layer, allowing bacteria to reach the epithelium and trigger inflammation; this illustrates that barrier integrity depends not only on the tight junctions but also on the chemical layer that overlies them.

## 3 The mucosal immune barrier

The intestinal barrier is not only a physical and chemical structure but also an immunological organ. The gut-associated lymphoid tissue (GALT) is the largest component of the immune system in the body and operates in close functional contact with the epithelium, so that the integrity of the barrier and the activity of the mucosal immune system are mutually dependent. When the paracellular pathway opens and luminal antigens reach the lamina propria in excess, the resulting immune activation is a central mechanism linking increased permeability to chronic inflammatory and autoimmune disease (3, 15, 71). The multi-layered organization of this interface is summarized in Table 2.

**Table 2:** The multi-layered organization of the intestinal mucosal barrier.

Barrier component	Principal elements	Main protective role
Physical barrier	Tight junctions, adherens junctions, desmosomes	Seal the paracellular space and provide mechanical cohesion of the epithelium
Chemical barrier	MUC-2 mucus layer (goblet cells); defensins and lysozyme (Paneth cells)	Limit direct contact between microorganisms and the epithelial surface
Immunological barrier	GALT, Peyer patches, M cells, dendritic cells, secretory IgA	Sample and exclude luminal antigens; maintain tolerance to food and commensals
Microbial barrier	Commensal gut microbiota and its metabolites (e.g., short-chain fatty acids)	Compete with pathogens, reinforce tight junctions and calibrate immune responses

GALT, gut-associated lymphoid tissue; IgA, immunoglobulin A.

### 3.1 Organized lymphoid tissue and antigen sampling

Organized lymphoid structures, including the Peyer patches of the small intestine and isolated lymphoid follicles, are covered by a specialized follicle-associated epithelium containing microfold (M) cells. M cells transcytose luminal antigens and microorganisms to the underlying dendritic cells, allowing controlled sampling of the luminal content without compromising the barrier. Dendritic cells can also extend dendrites between epithelial cells to sample antigens directly from the lumen. The way in which an antigen is presented, and the cytokine environment in which presentation occurs, determines whether the mucosal immune response is one of tolerance or of active defense (2, 6, 71).

### 3.2 Secretory immunoglobulin A

Secretory immunoglobulin A (sIgA) is the principal effector of humoral immunity at the mucosal surface and the most abundant antibody isotype produced in the body. Plasma cells in the lamina propria secrete dimeric IgA, which is transported across the epithelium by the polymeric immunoglobulin receptor and released into the lumen bound to the secretory component. There, sIgA binds bacteria, viruses, toxins and dietary antigens, preventing their adhesion to and penetration of the epithelium in a process known as immune exclusion, while generally avoiding the induction of inflammation. sIgA also shapes the composition and spatial organization of the microbiota, reinforcing the link between immune function and barrier homeostasis. A defective sIgA response can therefore increase the antigenic and microbial load reaching the epithelium and contribute to barrier dysfunction.

### 3.3 Tolerance, microbiota and immune homeostasis

Under physiological conditions the mucosal immune system maintains a state of controlled tolerance toward dietary antigens and commensal bacteria, mediated in large part by regulatory T cells and tolerogenic dendritic cells. The gut microbiota is essential for the correct development and calibration of this system: commensal bacteria stimulate the production of sIgA and antimicrobial peptides, promote the differentiation of regulatory T cells, and reinforce tight junctions, in part through metabolites such as short-chain fatty acids derived from the fermentation of dietary fiber. Dysbiosis, by contrast, can shift the balance toward a pro-inflammatory state, increase zonulin release and weaken the barrier. This bidirectional relationship between the microbiota, the barrier and the mucosal immune system explains why dietary factors that alter the microbiota also affect immune tolerance, and is the conceptual axis that connects the structural, immunological and nutritional sections of this review (3, 29, 71, 72) (Figure 1).

**Figure 1:**
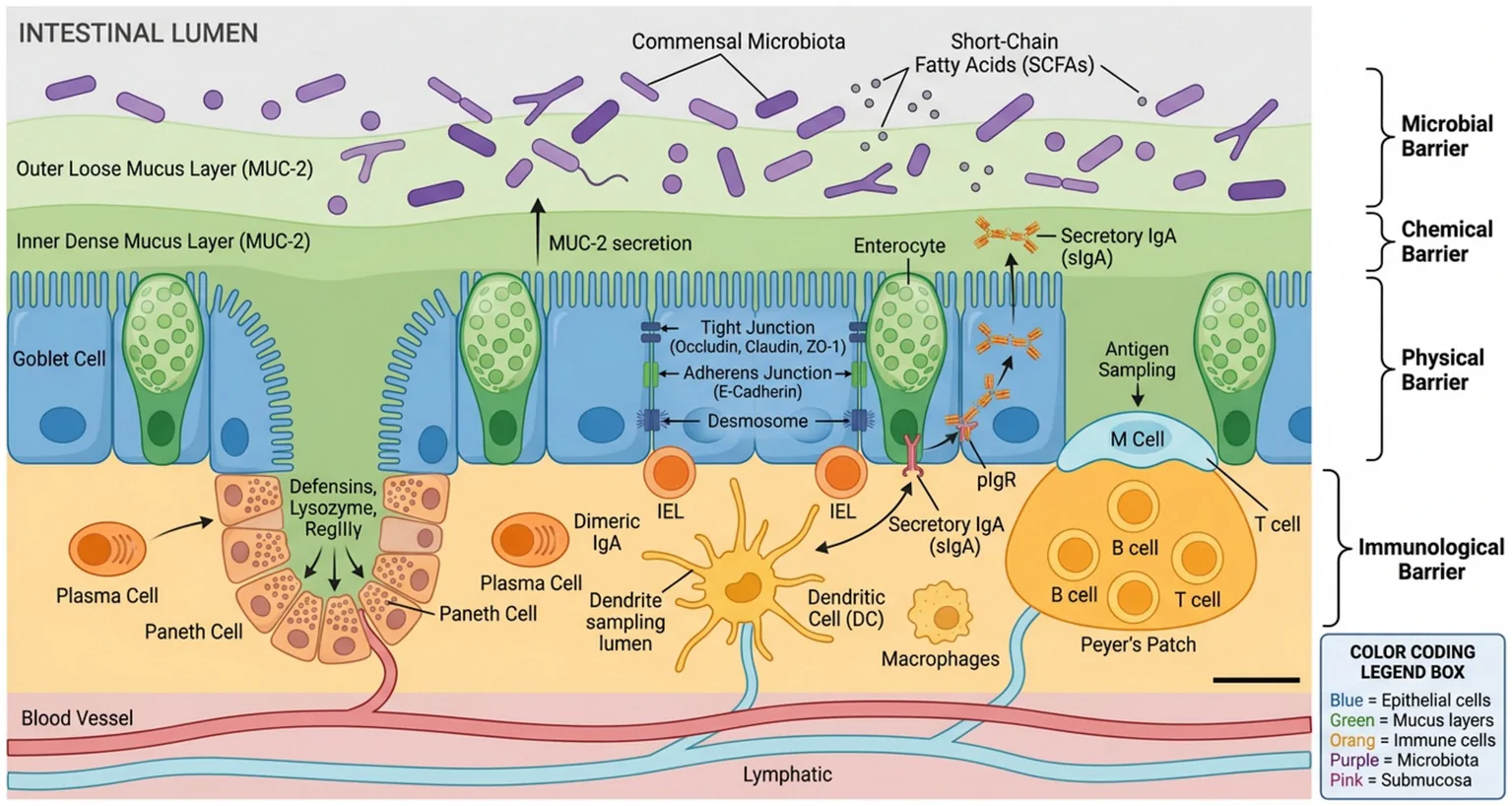
Multi-layered organization of the intestinal mucosal barrier. The intestinal barrier comprises four integrated layers: a microbial barrier (commensal microbiota producing short-chain fatty acids), a chemical barrier (MUC-2 mucus secreted by goblet cells; antimicrobial peptides released by Paneth cells), a physical barrier (enterocytes connected by tight junctions—occludin, claudin, ZO-1 —, adherens junctions and desmosomes), and an immunological barrier (secretory IgA, dendritic cells, M cells overlying Peyer’s patches, and the gut-associated lymphoid tissue). IEL, intraepithelial lymphocyte; pIgR, polymeric immunoglobulin receptor; sIgA, secretory immunoglobulin A; SCFAs, short-chain fatty acids.

## 4 Zonulin: the only known physiological modulator of tight junctions

One of the most important and often overlooked functions of the gastrointestinal tract is to provide a dynamic barrier that tightly controls antigen trafficking through both the transcellular and paracellular routes. The intercellular tight junctions are the key structures regulating paracellular trafficking of macromolecules, and zonulin is the only human protein discovered to date that reversibly regulates intestinal permeability by modulating these junctions; it was identified and characterized in the year 2000 by the research group of Fasano (23, 39).

In the 1990s a new toxin secreted by *Vibrio cholerae*, the zonula occludens toxin (Zot), was described. This toxin interacts with a specific cell-surface receptor present in the intestine (73) and brain (74) and induces protein kinase C alpha (PKC-alpha)-dependent polymerization of actin microfilaments, thereby regulating tight junctions and increasing the permeability of the epithelial layer (21, 75, 76). Through proteomic analysis of human serum, zonulin was subsequently identified as pre-haptoglobin-2 (19, 40). These data indicate that this multifunctional protein, in its intact single-chain form (zonulin), regulates intestinal permeability through transactivation of the epidermal growth factor receptor (EGFR) via protease-activated receptor 2 (PAR2), whereas in its two-chain form it acts as a hemoglobin scavenger (40).

The principal trigger of intestinal zonulin release is the C-X-C chemokine receptor type 3 (CXCR3) (77, 78), an inflammatory chemokine receptor whose expression is increased in the intestine of patients with coeliac or inflammatory bowel disease; luminal agents, including microorganisms and nutritional molecules, can also activate CXCR3-dependent zonulin release. The fundamental impact of the microbiota on zonulin release has been described as well: bacterial lipopolysaccharide (LPS) derived from *Escherichia coli* induces zonulin release in colonic epithelial cells, and gliadin fragments structurally resemble certain parasitic proteins that bind CXCR3 (79, 80).

The link between the gut microbiota and zonulin release is now well established, and several bacterial taxa have been shown to raise or lower zonulin levels. Genera reported to increase zonulin include Escherichia, Bacteroides, Prevotella, Pseudomonas, Shigella, Clostridium, *Ruminococcus gnavus* and Salmonella, whereas Corynebacterium, Bifidobacterium, Lactobacillus, Ruminococcus, Faecalibacterium and Lactococcus have been reported to decrease it.

### 4.1 The zonulin signaling pathway

At rest, tight-junction proteins are held in homophilic and heterophilic protein–protein interactions that keep the junctions fully closed. Upon activation, zonulin transactivates the EGFR via PAR2; phospholipase C is then activated and hydrolyzes phosphatidylinositol-4,5-bisphosphate to release inositol-1,4,5-trisphosphate (IP3) and diacylglycerol (DAG). Subsequently, PKC-α is activated directly by DAG, together with intracellular calcium release mediated by IP3 (81, 82). Activated, membrane-associated PKC-alpha catalyzes the phosphorylation of target proteins (including zonula occludens, cingulins and myosin) and the polymerization of soluble G-actin into F-actin. The combination of phosphorylation and actin polymerization rearranges the actin filaments and displaces the tight-junction complex proteins, so that the junction loses strength; once zonulin signaling ends, the tight junction returns to its closed state (72) (Figure 2).

**Figure 2:**
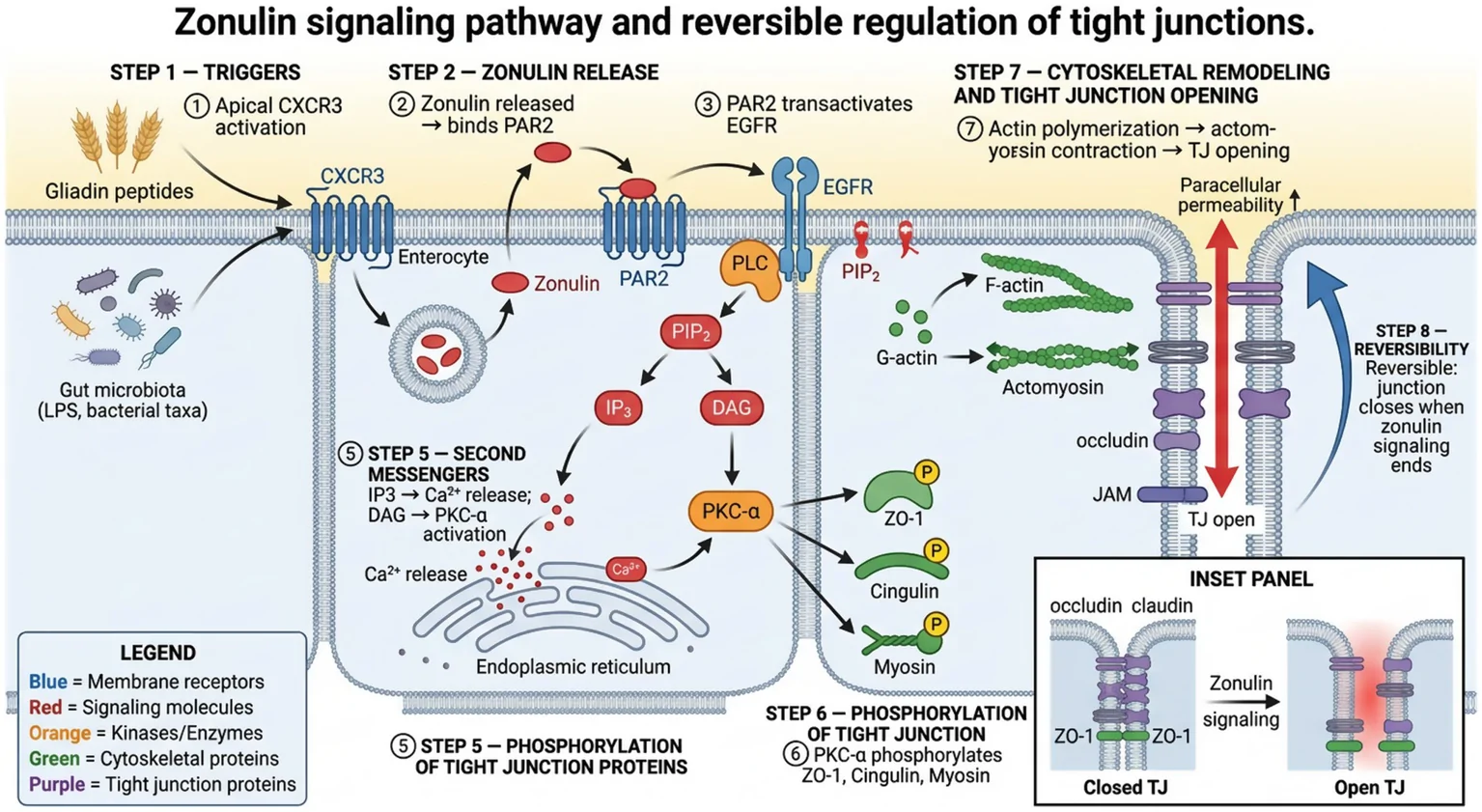
Zonulin signaling pathway and reversible regulation of tight junctions. Luminal triggers—gliadin peptides and gut microbiota-derived signals (LPS, bacterial taxa)—activate the apical CXCR3 receptor, inducing zonulin release. Zonulin binds PAR2, which transactivates EGFR; phospholipase C (PLC) then hydrolyzes PIP_2_ into IP_3_ and DAG. IP_3_-mediated Ca^2+^ release and DAG activate PKC-α, which phosphorylates ZO-1, cingulin and myosin, driving G-actin to F-actin polymerization and actomyosin contraction. The resulting displacement of occludin and claudin opens the paracellular space. The process is reversible: tight junctions return to the closed state once zonulin signaling ends (inset). DAG, diacylglycerol; EGFR, epidermal growth factor receptor; IP_3_, inositol-1,4,5-trisphosphate; JAM, junctional adhesion molecule; PAR2, protease-activated receptor 2; PIP_2_, phosphatidylinositol-4,5-bisphosphate; PKC-α, protein kinase C alpha; PLC, phospholipase C; ZO-1, zonula occludens-1.

## 5 Dietary factors that compromise intestinal barrier integrity

Beyond gliadin and the microbiota, a number of dietary components increase intestinal permeability, generally through a combination of microbiota disruption (dysbiosis), local inflammation and oxidative stress, and direct effects on tight-junction proteins. Because these factors act largely through the diet-microbiota-barrier-immunity axis described above, they are of particular interest from a nutritional-immunology perspective. The principal factors are summarized below and in Table 3.

**Table 3:** Dietary factors associated with increased intestinal permeability and their principal mechanisms.

Dietary factor	Principal mechanisms reported
Excess glucose	Retrograde GLUT-2 transport reducing junction-protein glycosylation; SGLT-1/MLCK activation; dysbiosis; reduced ZO and occludins
Excess LPS	TLR4/NF-kappaB activation; increased IL-6, TNF-alpha and nitric oxide; incorporation into chylomicrons; metabolic endotoxemia
Excess fructose	Uncontrolled fructokinase activity; ATP depletion and uric-acid-driven oxidative stress; reduced ZO and occludins; dysbiosis
Excess fat	Bile-acid-driven EGFR autophosphorylation and SRC-dependent occludin dephosphorylation and redistribution; reduced occludin, claudin-1, claudin-3 and JAM-1 expression; chylomicron accumulation in the lateral intercellular space proposed, but not demonstrated, to weaken junctions; dysbiosis
Omega-6 fatty acids	Arachidonic-acid eicosanoids (leukotrienes via CysLT1R, prostaglandins via EP receptors) activating MLCK and redistributing occludins and claudins
Excess salt	SGLT-1 and NHE3 activation; myosin light-chain phosphorylation; dysbiosis; altered epithelial proliferation
Alcohol	Acetaldehyde-induced oxidative stress; disassembly of tight-junction proteins; reduced MUC-2; stem-cell damage; dysbiosis and translocation
Food additives	Emulsifiers and artificial sweeteners damaging tight-junction proteins, reducing MUC-2, promoting inflammation and dysbiosis

EGFR, epidermal growth factor receptor; LPS, lipopolysaccharide; MLCK, myosin light-chain kinase; NHE3, Na+/H+ exchanger 3; ZO, zonula occludens.

### 5.1 Excess glucose

Hyperglycemia is an important driver of intestinal permeability, because high serum glucose disrupts the tight junctions between enterocytes through two mechanisms. First, retrograde transport of glucose into the enterocyte via GLUT-2 reduces the expression of genes involved in the glycosylation of proteins responsible for cell stability, including tight-junction proteins. Second, high luminal glucose can pass paracellularly and, by stimulating the SGLT-1 cotransporter, activates myosin light-chain kinase (MLCK), which phosphorylates myosin, induces contraction of the cytoskeletal filaments and rearranges junction proteins, increasing paracellular permeability (22, 83). A high-glucose diet also promotes dysbiosis, with a reported increase in proteolytic microbiota and a decrease in protective, saccharolytic microbiota, accompanied by reduced expression of zonula occludens and occludins (84).

### 5.2 Excess lipopolysaccharides and metabolic endotoxemia

Changes in the microbiota that favor Gram-negative bacteria raise circulating LPS and the risk of endotoxemia. LPS interacts with LPS-binding protein (LBP) on enterocytes or immune cells, activating Toll-like receptor 4 (TLR4) and NF-kappaB, which increases the release of pro-inflammatory cytokines such as IL-6 and TNF-alpha and of oxidant agents such as nitric oxide; this inflammatory and oxidative process disturbs tight-junction protein synthesis and permeability (85–87). LPS can also be incorporated into chylomicrons through its lipid tail, promoting endotoxemia under conditions of high intestinal permeability (88). In people with obesity, serum endotoxins and increased zonulin have been reported after a high-fat meal, suggesting that high fat intake influences barrier alterations (89).

### 5.3 Excess fructose

Fructose consumption has risen sharply, particularly as high-fructose corn syrup in ultra-processed foods. Intestinal fructokinase lacks feedback inhibition, so it cannot stop its own activity; the reaction consumes large amounts of ATP and stimulates adenosine deaminase, leading to degradation of adenine nucleotides and intracellular uric acid production, a molecule capable of generating oxidative stress (90, 91). Increased intestinal fructokinase expression in mice is accompanied by reduced tight-junction protein expression and increased permeability (92). High fructose intake has also been linked to pro-inflammatory responses and dysbiosis, with reduced zonula occludens and occludin expression and raised serum endotoxin (93–96).

### 5.4 Excess dietary fat

A high-fat diet is directly associated with alterations of barrier integrity, through dysbiosis and through altered intestinal absorption and lipid metabolism. High fat intake can lead to an uncontrolled presence of bile acids (deoxycholic, chenodeoxycholic and taurocholic acid) in the lumen, promoting tight-junction disruption. In Caco-2 monolayers, micromolar concentrations of deoxycholic and chenodeoxycholic acid induce autophosphorylation of the EGFR and, through an SRC-dependent step, dephosphorylation and redistribution of occludin away from the tight junction, with a reversible fall in transepithelial electrical resistance; co-incubation with an anti-EGF antibody or a specific SRC inhibitor abolishes the effect (97). Consistent with these *in vitro* findings, feeding rats a high-fat diet raises plasma bile acid concentrations and lowers the expression of occludin, claudin-1, claudin-3 and junctional adhesion molecule-1 in the small intestine, and exposure of Caco-2 monolayers to either rat bile juice or a fat emulsion reproduces both the permeability increase and the loss of junction proteins (98). A second mechanism has been proposed on morphological grounds: chylomicrons secreted across the basolateral membrane accumulate in the lateral intercellular space before crossing the basement membrane (99), and this distension has been argued to impose physical stress on the overlying tight junctions and thereby contribute to increased permeability (100). Direct evidence that such pressure alters paracellular flux is currently lacking, however, and this proposal should be regarded as a hypothesis rather than an established mechanism.

### 5.5 Excess omega-6 fatty acids

Omega-6 polyunsaturated fatty acids tend to induce pro-inflammatory responses. Arachidonic acid, the best-studied omega-6 fatty acid, is metabolized by intestinal epithelial cells into eicosanoids: increased leukotrienes bind the CysLT1 receptor and activate MLCK, while increased prostaglandins interact with prostaglandin E receptors, both leading to redistribution of tight-junction proteins such as occludins and claudins and to increased paracellular permeability (99, 101–103). Because omega-6 intake is currently high, mainly through processed products, it may contribute to chronic barrier dysfunction.

### 5.6 Excess salt, alcohol and food additives

High-salt diets can cause dysbiosis and affect epithelial cells; sodium stimulates SGLT-1 and activates the Na+/H + exchanger NHE3, leading to myosin light-chain phosphorylation, contraction of the actomyosin ring and increased permeability (104–107). Alcohol is absorbed in the small intestine, and accumulation of its metabolite acetaldehyde promotes oxidative stress and disassembly of tight-junction proteins, reduces the MUC-2 mucus layer, damages intestinal stem cells and alters the microbiota, favoring bacterial translocation and endotoxemia (108–117). Among food additives, synthetic emulsifiers such as carboxymethylcellulose and polysorbate 80, and artificial sweeteners such as sucralose, acesulfame-K and saccharin, have been shown in animal and cell models to damage tight-junction proteins, reduce MUC-2 expression, promote inflammation and induce marked dysbiosis (118–130).

## 6 Dietary factors that support or restore intestinal barrier integrity

Not every dietary exposure damages the gut wall—a growing body of research has mapped compounds that act in the opposite direction, reinforcing tight junctions, curbing zonulin release and giving the epithelium the raw materials it needs to repair itself. These agents range from fermentation-derived metabolites to micronutrients and botanical extracts, and their study has opened a practical arm of mucosal biology: designing nutritional strategies that either preserve barrier integrity or restore it once compromised. Because all of these effects unfold within the diet-microbiota-barrier-immunity axis, they also bear directly on mucosal immune balance.

### 6.1 Short-chain fatty acids and dietary fiber

Short-chain fatty acids (SCFAs)—butyrate, propionate and acetate—are produced when commensal bacteria ferment dietary fiber anaerobically, and they serve as the main energy source for colonocytes. Of the three, butyrate has attracted the most mechanistic scrutiny. It raises the expression of occludin, claudin-1 and zonula occludens-1 by engaging the Wnt/β-catenin pathway and blocking histone deacetylases (HDACs); it also drives goblet-cell secretion of MUC-2 mucin and steers naive T cells in the lamina propria toward a regulatory phenotype that dampens excessive mucosal inflammation (23, 39, 40, 131, 132). Diets rich in fermentable fiber—legumes, whole grains, vegetables and fruit—translate these mechanisms into measurable clinical signals: they raise fecal SCFA concentrations, lower circulating zonulin and mucosal inflammation markers, and improve lactulose/mannitol ratios in human intervention trials (133, 134). The reverse is equally telling: low-fiber Western diets deplete keystone producers such as *Faecalibacterium prausnitzii* and *Roseburia intestinalis* and, with them, much of the microbiota’s barrier-supportive capacity.

### 6.2 Omega-3 polyunsaturated fatty acids

Where excess omega-6 fatty acids erode the intestinal barrier, long-chain omega-3 s appear to do the opposite. Eicosapentaenoic acid (EPA) and docosahexaenoic acid (DHA)—sourced chiefly from oily fish and algae—displace arachidonic acid at cyclooxygenase and lipoxygenase active sites, redirecting eicosanoid production away from pro-inflammatory prostaglandins and leukotrienes and toward pro-resolving mediators: resolvins, protectins and maresins (135, 136). At the junction level, DHA supplementation raises claudin-3 and occludin expression and prevents cytokine-driven redistribution of zonula occludens-1 in epithelial cell models; EPA, meanwhile, dampens NF-κB inflammatory signaling in the same compartment (137, 138). These molecular observations have clinical correlates: in controlled human trials, omega-3 supplementation blunts the permeability increases provoked by both endurance exercise and experimental endotoxin challenge (139).

### 6.3 Polyphenols

A diverse class of plant-derived polyphenols—quercetin, resveratrol, curcumin, epigallocatechin gallate (EGCG) and anthocyanins among them—has been studied for barrier-protective activity, though the mechanistic detail available for each compound varies considerably. Quercetin, found at relatively high concentrations in onions, capers and apples, anchors occludin and zonula occludens-1 at the membrane and reduces zonulin secretion by blocking CXCR3 signaling (140, 141). Curcumin targets both the NF-κB and MLCK pathways through which cytokines force junction opening, and has been shown to lower LPS-induced permeability in rodent metabolic-syndrome models (142, 143). Resveratrol operates partly through SIRT1 activation—the resulting deacetylation stabilizes tight-junction scaffolding proteins—and partly by shifting the microbiota toward higher Lactobacillus and Bifidobacterium proportions (144, 145). A caveat worth noting: the small intestine absorbs most polyphenols poorly, so their principal actions probably occur in the colon, where microbial transformation generates bioactive metabolites that may differ substantially from the parent compound.

### 6.4 Zinc

Zinc carries structural and catalytic responsibilities in over 300 enzymatic reactions, but its relevance to epithelial biology runs deeper than mere enzyme-cofactor status. The element holds cysteine-rich zinc-finger domains within claudins and zonula occludens proteins in place, and withdrawing zinc from these sites causes a rapid and measurable rise in paracellular permeability in both cell cultures and whole-animal models (146, 147). Randomized trials in zinc-deficient children document falls in intestinal permeability and diarrhea incidence with supplementation, and animal work extends these findings to alcohol- and NSAID-induced barrier disruption (148, 149). From a public-health angle, mild-to-moderate zinc deficiency is far from rare—it affects many populations subsisting on low-quality diets and is disproportionately common in older adults—which makes adequate zinc intake a practical, achievable target for supporting barrier health.

### 6.5 Vitamin D

Vitamin D’s route into intestinal barrier biology runs through the nuclear vitamin D receptor (VDR), expressed in epithelial cells throughout the gut. When activated, VDR drives transcription of claudin-2, claudin-12, occludin and zonula occludens-1—a pattern that tightens the paracellular seal—while at the same time boosting Paneth-cell output of cathelicidin (LL-37) and β-defensin-2 and reducing NF-κB inflammatory signaling in the epithelium (150–152). Epidemiological data add another layer: low serum 25-hydroxyvitamin D is repeatedly associated with elevated circulating zonulin and heightened permeability markers across conditions as different as inflammatory bowel disease, type 1 diabetes and multiple sclerosis (153, 154). Given that vitamin D insufficiency—conventionally defined as 25(OH)D below 50 nmol/L—is widespread across temperate latitudes and in indoor-dwelling populations, the clinical relevance of this relationship is hard to dismiss; supplementation trials in Crohn disease and irritable bowel syndrome report measurable gains in barrier biomarkers alongside symptom improvements (155).

### 6.6 L-Glutamine

L-Glutamine is the most plentiful free amino acid in circulation and the preferred energy substrate for rapidly dividing enterocytes. Ordinarily the gut draws heavily on systemic glutamine, but that supply can be outstripped during critical illness, sustained intense exercise or severe caloric restriction; when it is, the epithelium shifts into a catabolic state and barrier integrity declines (156). At the molecular level, glutamine drives tight-junction protein expression via the PI3K/Akt pathway, attenuates MLCK-dependent junction opening and donates substrates for glutathione synthesis—giving epithelial cells a meaningful antioxidant reserve (156, 157). The translational evidence is clearest in high-demand settings: enteral glutamine supplementation in surgical and critically ill patients has reduced intestinal permeability, bacterial translocation and infection rates across multiple randomized controlled trials, cementing its status as conditionally essential during metabolic stress (157).

### 6.7 Probiotics and prebiotics

Probiotic strains—primarily from Lactobacillus and Bifidobacterium but extending to Saccharomyces boulardii and *Akkermansia muciniphila*—shore up the intestinal barrier by several distinct routes: competitive exclusion of pathogens, stimulation of goblet-cell MUC-2 mucin and epithelial sIgA production, raised tight-junction protein expression, and suppressed zonulin release. *Akkermansia muciniphila* deserves particular mention: it thickens the mucosal layer and reduces paracellular permeability, and its relative abundance in the microbiome is inversely associated with circulating zonulin and metabolic-endotoxemia markers in human cohorts (48, 49, 158, 159). Prebiotic fibers—inulin, fructooligosaccharides, galactooligosaccharides and resistant starch—extend this picture by selectively feeding SCFA-producing and mucus-reinforcing taxa, amplifying the effects discussed in the SCFAs subsection above. Combining a probiotic with a matching prebiotic in a synbiotic formulation yields additive improvements in barrier biomarkers; this has been documented in clinical trials covering irritable bowel syndrome, obesity and type 2 diabetes (160, 161). The principal protective dietary factors and their proposed mechanisms are brought together in Table 4.

**Table 4:** Dietary factors reported to support or restore intestinal barrier integrity and their principal mechanisms.

Dietary factor	Principal mechanisms reported
Short-chain fatty acids and dietary fiber	Butyrate raises occludin, claudin-1 and ZO-1 expression via Wnt/beta-catenin signaling and HDAC inhibition; stimulates goblet-cell MUC-2 secretion; promotes regulatory T-cell phenotypes in the lamina propria; fermentable fiber raises fecal SCFAs, lowers circulating zonulin and improves lactulose/mannitol ratios
Omega-3 polyunsaturated fatty acids	EPA and DHA displace arachidonic acid at COX and LOX, redirecting eicosanoid synthesis toward resolvins, protectins and maresins; DHA raises claudin-3 and occludin and prevents cytokine-driven ZO-1 redistribution; EPA dampens epithelial NF-kappaB signaling
Polyphenols	Quercetin anchors occludin and ZO-1 at the membrane and reduces zonulin secretion through CXCR3 blockade; curcumin targets NF-kappaB and MLCK signaling; resveratrol acts via SIRT1-dependent stabilization of junction scaffolds and microbiota modulation; principal actions probably colonic owing to poor small-intestinal absorption
Zinc	Stabilizes cysteine-rich zinc-finger domains within claudins and ZO proteins; withdrawal raises paracellular permeability in cell and animal models; supplementation lowers permeability and diarrhea incidence in zinc-deficient children
Vitamin D	VDR-dependent transcription of claudin-2, claudin-12, occludin and ZO-1; increased Paneth-cell cathelicidin (LL-37) and beta-defensin-2 output; reduced epithelial NF-kappaB signaling
L-Glutamine	Preferred energy substrate for enterocytes; drives tight-junction protein expression via PI3K/Akt; attenuates MLCK-dependent junction opening; donates substrate for glutathione synthesis
Probiotics and prebiotics	Competitive exclusion of pathogens; stimulation of goblet-cell MUC-2 and epithelial secretory IgA; raised tight-junction protein expression; suppressed zonulin release; *Akkermansia muciniphila* thickens the mucus layer; prebiotic fibers selectively feed SCFA-producing and mucus-reinforcing taxa

COX, cyclooxygenase; CXCR3, C-X-C chemokine receptor 3; DHA, docosahexaenoic acid; EPA, eicosapentaenoic acid; HDAC, histone deacetylase; LOX, lipoxygenase; MLCK, myosin light-chain kinase; SCFA, short-chain fatty acid; VDR, vitamin D receptor; ZO, zonula occludens.

## 7 Clinical conditions associated with barrier dysfunction

Increased intestinal permeability and elevated zonulin have been reported across a wide and growing range of conditions. The associations are strongest, and most mechanistically plausible, within the gastrointestinal tract, but a large body of work has extended them to extra-intestinal and systemic disease. The following paragraphs summarize the main groups of conditions; in most cases the available evidence is associative and does not establish causality.

### 7.1 Gastrointestinal and hepatic conditions

Within the gastrointestinal tract, increased permeability and raised zonulin have been described in coeliac disease, non-coeliac gluten sensitivity, irritable bowel syndrome, inflammatory bowel disease, necrotizing enterocolitis and environmental enteric dysfunction (142–144). In coeliac disease in particular, gliadin is a direct trigger of zonulin release, providing a coherent mechanistic link between a dietary antigen, barrier opening and immune activation. Beyond the gut, raised zonulin has been reported in several liver diseases, including non-alcoholic fatty liver disease, hepatitis, cirrhosis and hepatocellular carcinoma, where increased permeability may allow pathogens, antigens and toxic metals to reach the liver through the portal circulation and trigger inflammatory responses (142–144).

### 7.2 Metabolic, hormonal and autoimmune conditions

Elevated zonulin and barrier dysfunction have been linked to metabolic and hormonal disorders, including type 1 and type 2 diabetes, obesity, insulin resistance and polycystic ovary syndrome (145–154). In these conditions, translocation of bacterial products such as LPS and the resulting low-grade systemic inflammation (metabolic endotoxemia) are thought to contribute to insulin resistance. Barrier dysfunction has also been associated with autoimmune diseases such as rheumatoid arthritis, ankylosing spondylitis and Hashimoto thyroiditis, where barrier integrity may determine the severity of systemic inflammation through the migration of immune cells from the gut to target organs (134, 162–166), consistent with the hypothesis that loss of barrier control is a permissive factor for autoimmunity in genetically susceptible individuals.

### 7.3 Neuropsychiatric, viral and cardiovascular conditions

A range of neuropsychiatric and neurological conditions has been associated with barrier dysfunction, including attention-deficit/hyperactivity disorder, autism spectrum disorder, bipolar disorder, anxiety and depression, Alzheimer disease, Parkinson disease and multiple sclerosis (41, 167–175). Brain endothelial cells express the zonulin receptors EGFR and PAR2, and exposure of the blood–brain barrier to zonulin increases its permeability (176, 177), offering a potential mechanism linking intestinal and central-nervous-system barriers within the gut-brain axis. Associations have additionally been reported with viral infections, including SARS-CoV-2, HIV and hepatitis B, where sustained high zonulin has been linked to more severe outcomes and neurological involvement, and with cardiovascular disease (178–184).

## 8 Laboratory assessment of intestinal permeability

Several tests allow direct or indirect evaluation of intestinal paracellular permeability, each with distinct advantages and limitations.

### 8.1 Lactulose/mannitol urinary test

The fasting patient ingests a solution containing two non-metabolized sugar alcohols of different size: lactulose or lactitol (large molecules, ~0.34 kDa) representing macronutrients that do not normally cross the barrier, and mannitol (a small molecule, ~0.18 kDa) representing micronutrients normally absorbed by the enterocyte microvilli. Urine is collected over the following 5 h. A higher-than-expected recovery of the large molecule indicates increased permeability, whereas a lower-than-expected recovery of mannitol indicates reduced absorptive surface, which may relate to malnutrition. The ratio of the two molecules corrects for confounders such as gastric emptying and renal function, but results are sensitive to collection protocol, which has limited comparability between studies (185).

### 8.2 Lipopolysaccharide (LPS) and LPS-binding protein (LBP)

LPS are large molecules (10–20 kDa) located in the outer membrane of Gram-negative bacteria and transported mainly by the transcellular route; increased levels are a sign of barrier dysfunction and of metabolic endotoxemia. Because LPS readily contaminates common laboratory equipment and is difficult to measure reliably, LBP is often used as a more stable, non-invasive surrogate marker of increased transcellular LPS absorption (186–190).

### 8.3 Intestinal fatty-acid-binding protein (I-FABP)

I-FABP is a cytosolic protein present in differentiated enterocytes of the small intestine and, to a lesser extent, the large intestine. Under normal conditions only small amounts circulate, but it is released into the bloodstream after damage to the intestinal barrier, making it a sensitive marker of enterocyte injury, although it reflects cell damage rather than paracellular permeability as such (191–195).

### 8.4 Circulating junction proteins and serum zonulin

A limited number of studies have reported increased systemic levels of transmembrane and scaffolding junction proteins, for example serum claudin in children with ADHD, urinary claudin in active Crohn disease, and zonula occludens-1 in fibromyalgia and chronic fatigue syndrome, as markers of altered barrier function, although these markers still require validation. Serum zonulin is the most widely used biomarker, but its interpretation warrants particular caution: the most commonly used commercial ELISA may not detect pre-haptoglobin-2 specifically and may recognize other proteins of the same family, so zonulin results should be interpreted alongside complementary measures rather than in isolation (196–198).

## 9 Critical appraisal of the evidence

### 9.1 Causality, consequence or epiphenomenon

The evidence assembled above establishes that increased intestinal permeability accompanies a remarkable range of conditions, but accompaniment is not causation. Three readings remain compatible with most published data: barrier failure as an initiating event, barrier failure as a downstream consequence of mucosal inflammation, and barrier failure as an epiphenomenon that tracks disease activity without contributing to it. The distinction is not academic, since only the first justifies barrier-directed intervention. Experimental work suggests that loss of barrier function is rarely sufficient on its own; it has been argued that isolated increases in permeability produce mucosal immune activation but not overt disease unless a second insult—a genetic susceptibility, a microbial trigger or an immune stimulus—is also present (199). Reverse causation is equally difficult to exclude, because the pro-inflammatory cytokines generated during active disease are themselves potent inducers of myosin light-chain kinase-dependent junction opening. Almost all of the human associations cataloged in the preceding sections derive from cross-sectional designs, in which the direction of the relationship cannot be determined; prospective data showing that permeability precedes clinical onset remain confined to a small number of conditions.

### 9.2 Direct permeability testing versus indirect biomarkers

A recurrent source of inconsistency across studies is that “increased intestinal permeability” is operationalized in ways that are not equivalent. Dual- and multi-sugar urinary tests measure paracellular flux directly, whereas lipopolysaccharide-binding protein, intestinal fatty-acid-binding protein, circulating junction proteins and zonulin are indirect surrogates, each reflecting a different biological event: I-FABP indicates enterocyte injury rather than paracellular leak, and LBP reflects transcellular endotoxin exposure and hepatic acute-phase response. Correlations among these markers are generally modest, so studies using different readouts are not directly comparable, and discordant findings may reflect measurement rather than biology. Sugar tests themselves are sensitive to probe dose, collection window and renal function, which has limited comparability between laboratories. Serum zonulin, the most widely used marker in the literature reviewed here, carries the largest uncertainty: the commercial immunoassays on which most published values rest have been shown not to detect pre-haptoglobin-2 specifically, recognizing instead properdin, haptoglobin or complement C3 (200). Much of the clinical zonulin literature must therefore be read as provisional.

### 9.3 Regional heterogeneity along the gastrointestinal tract

Barrier properties are not uniform along the gut. Claudin expression profiles, the relative contribution of pore and leak pathways, mucus thickness and microbial density all differ markedly between small intestine and colon, and dietary exposures act preferentially on one segment or the other: bile acids, fructose and fat operate mainly proximally, whereas fiber fermentation, short-chain fatty acid production and most polyphenol metabolism occur in the colon. Permeability testing can in principle resolve this, since early urine collections reflect small-intestinal and late collections colonic permeability (201), yet the majority of the studies reviewed here used a single collection window and therefore report a composite signal that cannot be attributed to a region. This limits the extent to which a mechanism demonstrated in one segment can be assumed to explain a permeability change measured across the whole tract.

### 9.4 Extrapolation from cell and animal models to human dietary exposure

Much of the mechanistic detail presented in this review derives from Caco-2 monolayers and rodent feeding studies, and the translational distance should not be understated. Cell models are typically challenged with concentrations of bile acids, fructose, emulsifiers or sweeteners that exceed those plausibly reached in the human intestinal lumen, and they lack mucus, microbiota and immune cells. Rodent high-fat diets commonly supply 45–60% of energy as fat, above habitual human intakes, and species differences in bile acid pool composition and microbial ecology further constrain extrapolation. Demonstrated mechanistic plausibility therefore does not establish that a given dietary component compromises the barrier at intakes achievable in a normal diet, and this caveat applies equally to the protective agents: most human evidence for fiber, omega-3 fatty acids, polyphenols, zinc, vitamin D, glutamine and probiotics comes from short supplementation trials with surrogate endpoints rather than from dietary patterns studied over clinically meaningful periods.

### 9.5 Controversies, research gaps and priorities

Three controversies dominate the field. The first is analytical and concerns the validity of commercial zonulin assays, which has consequences for the interpretation of a substantial part of the literature. The second is conceptual: whether “leaky gut” constitutes a clinical entity with diagnostic criteria, or a physiological variable that shifts across many diseases without defining any of them. The third is therapeutic: whether barrier-directed nutritional intervention modifies disease course, or merely biomarkers. Progress on all three requires the same foundations, which are currently absent—validated permeability tests with regional resolution and established reference ranges, head-to-head comparison of candidate biomarkers against a direct measure in the same participants, and randomized trials of sufficient duration to capture clinical rather than surrogate endpoints. Dose–response studies at intakes achievable through diet, and characterization of responses in defined populations such as older adults, athletes and patients with established inflammatory disease, are the most immediate priorities.

### 9.6 Position of this review in relation to existing reviews

Existing reviews of intestinal permeability and zonulin generally address a subset of junction components, most often occludin, the claudins and zonula occludens-1. The specific contribution of the present review is to assemble the full complement of apical junctional proteins—including junctional adhesion molecules, tricellulin with its angulin partners, and the cingulins, which are frequently omitted—together with their reported clinical associations, and to connect them within a single framework to dietary exposures acting in both directions, damaging and protective. This integrated treatment is what allows the diet-microbiota-barrier-immunity axis to be considered as a whole rather than as a series of separate mechanisms. The corresponding limitation is one the field shares: beyond zonulin, whose measurement is itself contested, there are no validated diagnostic markers of intestinal permeability, and the central mechanism was only characterized in 2000, so the evidence base remains young and is still dominated by association studies.

## 10 Conclusion

The intestinal barrier is a dynamic, multi-layered and finely regulated interface whose integrity is essential for mucosal immune homeostasis. Tight junctions, supported by adherens junctions and desmosomes and overlaid by a chemical barrier of mucus and antimicrobial peptides, are the principal determinants of paracellular permeability, while the gut-associated lymphoid tissue, secretory IgA and a tolerogenic immune environment complete a defensive system in which barrier function and immune activity are mutually dependent. Zonulin remains the only endogenous protein known to modulate tight junctions reversibly, under the strong influence of gliadin and the gut microbiota. A consistent body of evidence links increased permeability and elevated zonulin to gastrointestinal, hepatic, metabolic, autoimmune, neuropsychiatric, viral and cardiovascular conditions, although in most cases the observed associations do not establish causality. Importantly from a nutritional perspective, several common dietary components (excess glucose, fructose, fat, omega-6 fatty acids, salt and alcohol, as well as specific food additives) can compromise barrier integrity, largely through dysbiosis, inflammation, oxidative stress and direct effects on junction proteins, and thereby modulate mucosal immune activation. A clearer understanding of this diet-microbiota-barrier-immunity axis, together with better-validated permeability biomarkers, may help to define nutritional strategies aimed at preserving intestinal barrier function. Well-designed human studies remain necessary to confirm these mechanisms and to translate them into dietary recommendations.

The evidence reviewed here sketches a two-sided dietary picture: certain compounds erode the barrier, others reinforce it. Among the protective agents, butyrate—generated from fermentable fiber by commensal bacteria—stands out for the depth of its mechanistic characterization: it raises tight-junction protein expression, promotes goblet-cell mucin output and steers lamina-propria T cells toward regulatory phenotypes. Long-chain omega-3 s, polyphenols including quercetin and curcumin, and the micronutrients zinc and vitamin D converge on overlapping molecular targets—NF-κB signaling, MLCK-dependent junction opening, CXCR3-mediated zonulin release—which argues for multicomponent rather than single-nutrient interventions. Probiotic and synbiotic combinations, particularly those incorporating *Akkermansia muciniphila* alongside Lactobacillus and Bifidobacterium strains with matched prebiotic substrates, add further layers of protection in early clinical data. Closing these gaps will require well-powered mechanistic and interventional studies that integrate multi-omics microbiota characterization, and such studies are ultimately what will convert the science of the diet-microbiota-barrier-immunity axis into actionable clinical guidance.
